# Supplementary material for: Local seed sourcing for sustainable forestry
Source: PLoS One. 2022 Dec 14;17(12):e0278866. doi: 10.1371/journal.pone.0278866 (PMC9750025; doi:10.1371/journal.pone.0278866)
Supplement: S1 Annex — (DOCX) [file pone.0278866.s001.docx]

S1 Annex. Niche modelling and climatic suitability of the species and genetic pools.

The niche modelling procedure and determination of the Wide-sense local species pools were described in [1]

*Environmental information*

The natural distribution has been transformed in presence/absence data using a 1 x 1 km grid. For each point of the grid, climate data corresponding to the (1961–1990 period) were obtained using two different climatic models for the Iberian Peninsula [2], and for the Balearic islands [3]. The variables included mean annual (P), winter (WP), and summer (SP) precipitation, mean annual temperature (T), minimum temperature of the coldest month (MTC), maximum temperature of the warmest month (MTW), growing degree days (t>5°) (DD), total number of months under frost (t<0º) (F), duration of the drought period in months (P<2T) (DP). These variables were chosen because of their strong link with the physiology and growth of plant species [4] and most of them have been used for modelling niche distribution of different forest species in Spain [5]. For instance, MTC discriminates species based on their ability to assimilate soil water and nutrients, and continue cell division, differentiation and tissue growth at low temperatures (lower limit), and chilling requirements for processes such as bud break and seed germination (upper limit). Altitude (ALT) was also considered.

*Niche-Based Models of Species Climatic Envelopes*

For each species following a maximum entropy modelling approach [6] using the Maxent Software was used to obtain the area climate-predictive for each of the different species. Species with less than 20 points were not considered - this was the case for three species. We obtained for each grid point the logistic probability of each species’ presence. As a background environmental file, we used half of the data grid points. The projection was made to all the points of the grid. We obtained the response curves for each variable, and the importance of each environmental variable (jacknife method).

*Wide-sense local pools (climatic similarity)*

To establish the assignment to the region of provenance, points with logistic probabilities of occurrence higher than 0.4 were considered. The probability of assignment to each of the regions of provenance was established according to a multi-normal distribution based on the same climatic variables. The average probability for each region of use and region of provenance was computed.

The wide-local pool for each species and deployment zone, was defined with those provenances with a suitable climatic niche in the deployment zone. Climatic information was obtained from the model developed by [2] at a resolution of 1x1 km2. Each region of provenance was classified according to the suitability in a given deployment zone: Highly suitable; Adequate; Possible use, Not suitable. This classification was based on the Mahalanobis climatic distance (using the same set of climatic variables that in the niche modelling estimation, see annex 1), among the points with presence of the Region of Provenance and the points of each of the deployment zone.

1. García del Barrio, J.M.; Auñón, F.; Sánchez de Ron, D.; Alía, R. Assessing regional species pools for restoration programs in Spain. *New For.* **2013**, *44*, 559–576, doi:10.1007/s11056-013-9363-y.

2. Gonzalo, J. *Diagnosis fitoclimática de la España Peninsular. Hacia un modelo de clasificacion funcional de la vegetación y de los ecosistemas peninsulares españoles*; Min. Medio Ambiene y Med. Rural y Marino: Madrid, 2010; ISBN 84-8014-787-3.

3. Hijmans, R.J.; Cameron, S.E.; Parra, J.L.; Jones, P.G.; Jarvis, A. The WorldClim interpolated global terrestrial climate surfaces 2004.

4. Bartlein, P.J.; Prentice, I.C.; Webb, T. Climatic Response Surfaces from Pollen Data for Some Eastern North American Taxa. *J. Biogeogr.* **1986**, *13*, 35, doi:10.2307/2844848.

5. Benito-Garzón, M.; Sánchez de Dios, R.; Sáinz Ollero, H. Predictive modelling of tree species distributions on the Iberian Peninsula during the Last Glacial Maximum and Mid-Holocene. *Ecography (Cop.).* **2007**, *30*, 120–134, doi:10.1111/j.2006.0906-7590.04813.x.

6. Phillips, S.J.; Anderson, R.P.; Schapire, R.E.; Schapired, R.E. Maximum entropy modeling of species geographic distributions. *Ecol. Modell.* **2006**, *190*, 231–259, doi:10.1016/j.ecolmodel.2005.03.026.
