# Supplementary material for: Local seed sourcing for sustainable forestry
Source: PLoS One. 2022 Dec 14;17(12):e0278866. doi: 10.1371/journal.pone.0278866 (PMC9750025; doi:10.1371/journal.pone.0278866)
Supplement: S1 Fig — Regiones de identificacion y utilizacion de material forestal de reproduccion. Serie Cartografica. Madrid: MAPA).BDLJE CC-BY 4.0 ign.es, DR miteco.gob.es. (DOCX) [file pone.0278866.s002.docx]

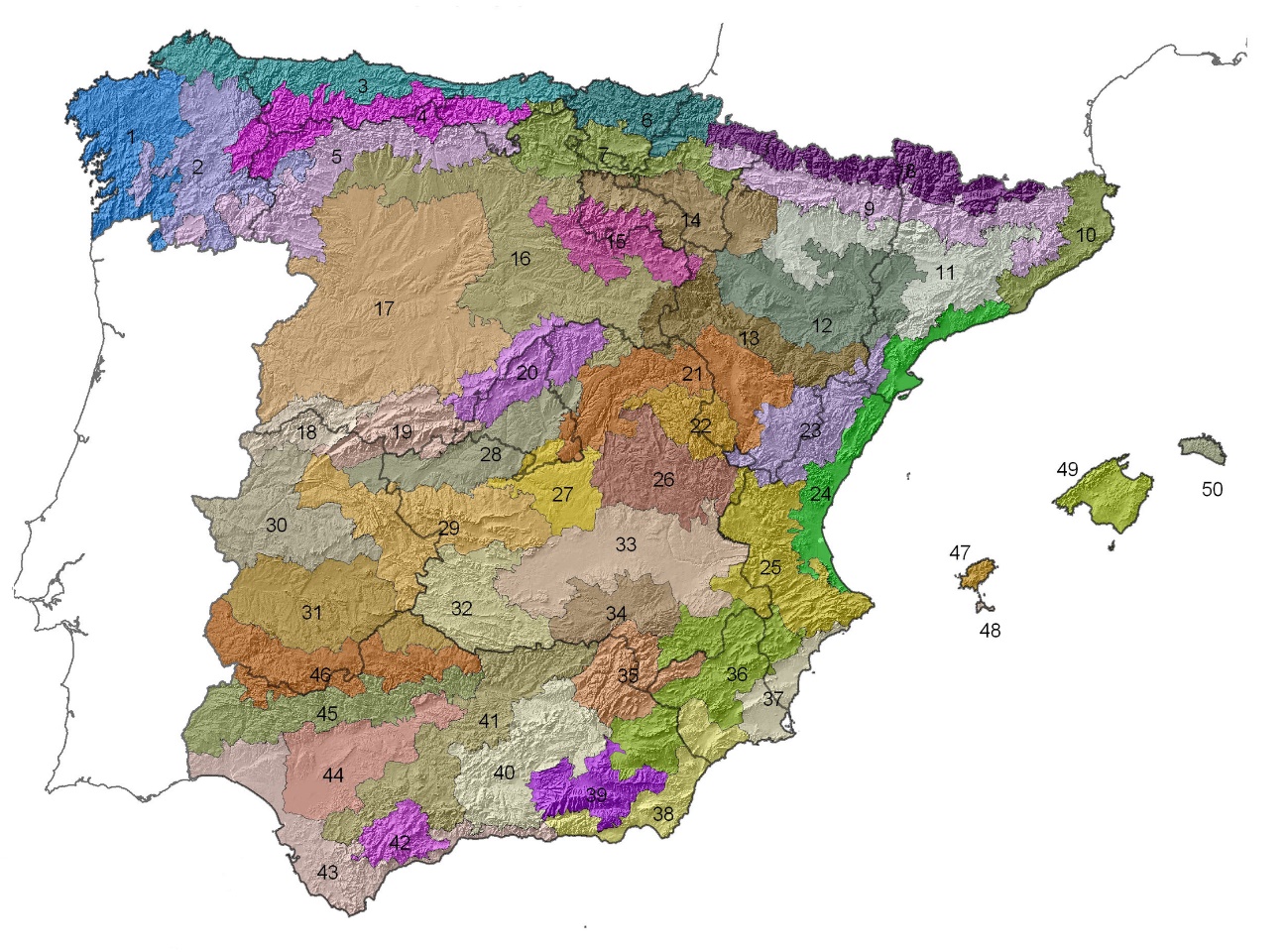


**Fig. S1**. Deployment regions defined in Spain (from García del Barrio et al. 2001. Regiones de identificacion y utilizacion de material forestal de reproduccion. Serie Cartografica. Madrid: MAPA). **BDLJE CC-BY 4.0 ign.es, DR miteco.gob.es**
