# Supplementary material for: Local seed sourcing for sustainable forestry
Source: PLoS One. 2022 Dec 14;17(12):e0278866. doi: 10.1371/journal.pone.0278866 (PMC9750025; doi:10.1371/journal.pone.0278866)
Supplement: S1 Table — (DOCX) [file pone.0278866.s003.docx]

**S1 Table.** Richness for the seed sourcing pools by deployment zone (DZ).

|  | **Strict-sense local pool** | | | | **Wide-sense local pool** | | | |
| --- | --- | --- | --- | --- | --- | --- | --- | --- |
| **DZ** | **Species** | **Available species** | **Genetic** | **Available genetic** | **Species** | **Available species** | **Genetic** | **Available genetic** |
| 1 | 18 | 7 | 21 | 8 | 31 | 20 | 58 | 26 |
| 2 | 20 | 12 | 22 | 14 | 35 | 28 | 145 | 70 |
| 3 | 25 | 10 | 33 | 13 | 32 | 26 | 131 | 65 |
| 4 | 24 | 19 | 37 | 25 | 37 | 33 | 175 | 112 |
| 5 | 25 | 21 | 40 | 35 | 37 | 33 | 146 | 101 |
| 6 | 29 | 21 | 43 | 31 | 35 | 28 | 98 | 64 |
| 7 | 31 | 23 | 48 | 36 | 37 | 34 | 200 | 128 |
| 8 | 33 | 23 | 55 | 38 | 38 | 32 | 132 | 86 |
| 9 | 35 | 23 | 63 | 42 | 39 | 32 | 141 | 84 |
| 10 | 36 | 14 | 42 | 18 | 38 | 31 | 141 | 59 |
| 11 | 34 | 12 | 50 | 23 | 38 | 26 | 129 | 56 |
| 12 | 12 | 5 | 18 | 11 | 19 | 12 | 67 | 28 |
| 13 | 21 | 12 | 25 | 16 | 30 | 24 | 157 | 71 |
| 14 | 27 | 17 | 39 | 27 | 35 | 31 | 164 | 97 |
| 15 | 29 | 22 | 33 | 25 | 36 | 31 | 157 | 92 |
| 16 | 26 | 22 | 45 | 40 | 34 | 31 | 225 | 141 |
| 17 | 24 | 19 | 37 | 29 | 31 | 29 | 168 | 103 |
| 18 | 19 | 12 | 24 | 17 | 30 | 26 | 137 | 71 |
| 19 | 21 | 19 | 27 | 25 | 29 | 26 | 158 | 81 |
| 20 | 29 | 21 | 39 | 30 | 34 | 30 | 245 | 120 |
| 21 | 20 | 8 | 27 | 15 | 29 | 24 | 194 | 94 |
| 22 | 24 | 16 | 27 | 19 | 31 | 26 | 104 | 51 |
| 23 | 26 | 17 | 35 | 25 | 36 | 31 | 180 | 86 |
| 24 | 25 | 11 | 39 | 18 | 29 | 12 | 45 | 25 |
| 25 | 22 | 12 | 31 | 18 | 29 | 25 | 194 | 89 |
| 26 | 23 | 13 | 28 | 18 | 28 | 25 | 119 | 59 |
| 27 | 10 | 6 | 12 | 7 | 19 | 16 | 76 | 39 |
| 28 | 17 | 11 | 26 | 18 | 27 | 23 | 145 | 63 |
| 29 | 22 | 14 | 28 | 20 | 28 | 20 | 198 | 72 |
| 30 | 13 | 6 | 17 | 9 | 18 | 12 | 44 | 20 |
| 31 | 14 | 7 | 17 | 9 | 18 | 13 | 43 | 19 |
| 32 | 15 | 9 | 18 | 12 | 22 | 17 | 105 | 44 |
| 33 | 17 | 10 | 24 | 17 | 22 | 18 | 116 | 52 |
| 34 | 17 | 10 | 19 | 12 | 23 | 20 | 110 | 50 |
| 35 | 21 | 13 | 26 | 18 | 27 | 21 | 122 | 62 |
| 36 | 19 | 11 | 32 | 19 | 22 | 17 | 92 | 42 |
| 37 | 9 | 4 | 11 | 5 | 16 | 10 | 34 | 18 |
| 38 | 12 | 6 | 19 | 10 | 21 | 17 | 95 | 44 |
| 39 | 22 | 10 | 24 | 12 | 28 | 19 | 106 | 39 |
| 40 | 22 | 9 | 25 | 12 | 28 | 17 | 132 | 52 |
| 41 | 18 | 12 | 26 | 19 | 23 | 16 | 118 | 52 |
| 42 | 19 | 9 | 23 | 12 | 24 | 16 | 68 | 30 |
| 43 | 18 | 14 | 30 | 21 | 21 | 16 | 58 | 35 |
| 44 | 12 | 8 | 16 | 11 | 13 | 9 | 34 | 19 |
| 45 | 14 | 9 | 19 | 13 | 18 | 13 | 53 | 26 |
| 46 | 14 | 9 | 18 | 11 | 19 | 13 | 46 | 20 |
| 47 | 5 | 1 | 5 | 1 | 11 | 3 | 21 | 4 |
| 48 | 4 | 1 | 4 | 1 | 8 | 3 | 10 | 4 |
| 49 | 13 | 2 | 13 | 2 | 23 | 6 | 44 | 9 |
| 50 | 7 | 1 | 7 | 1 | 10 | 6 | 24 | 6 |
