# Supplementary material for: Local seed sourcing for sustainable forestry
Source: PLoS One. 2022 Dec 14;17(12):e0278866. doi: 10.1371/journal.pone.0278866 (PMC9750025; doi:10.1371/journal.pone.0278866)
Supplement: S2 Table — (DOCX) [file pone.0278866.s004.docx]

**S2 Table.** Richness of seed sourcing pools and deployment zone for 40 forest tree species in Spain.

| **Species** | **Strict-sense local** | | | | | **Wide-sense local** | | | | |
| --- | --- | --- | --- | --- | --- | --- | --- | --- | --- | --- |
| **Code** | **Tgp_loc** | **Agp_loc** | **Tdr_loc** | **Edr_loc** | **Adr-loc** | **Tgp_cloc** | **Agp_cloc** | **Tdr_cloc** | **Edr_cloc** | **Adr-cloc** |
| aal | 3.3 | 2.7 | 3 | 1.2 | 3 | 1.8 | 1.8 | 10 | 1.6 | 10 |
| api | 2.0 | 2.0 | 2 | 1.1 | 2 | 1.5 | 1.5 | 6 | 1.5 | 6 |
| apl | 1.0 | 0.0 | 7 | 1.9 | 0 | 2.1 | 0.0 | 16 | 8.7 | 0 |
| aps | 1.0 | 0.5 | 19 | 4.7 | 10 | 3.4 | 2.4 | 21 | 8.3 | 18 |
| aun | 1.0 | 0.6 | 47 | 15.0 | 28 | 4.9 | 3.1 | 50 | 26.0 | 47 |
| bpu | 1.0 | 0.6 | 23 | 5.4 | 14 | 5.5 | 2.9 | 24 | 6.9 | 19 |
| csa | 1.0 | 0.5 | 37 | 8.0 | 20 | 5.3 | 2.5 | 42 | 8.9 | 40 |
| fsy | 3.1 | 3.1 | 16 | 7.0 | 16 | 2.9 | 2.9 | 18 | 8.2 | 18 |
| fex | 1.0 | 0.6 | 17 | 6.8 | 11 | 5.3 | 2.9 | 19 | 7.8 | 18 |
| iaq | 1.0 | 0.7 | 30 | 9.7 | 20 | 5.7 | 3.3 | 31 | 10.7 | 28 |
| jre | 1.0 | 0.4 | 39 | 18.0 | 15 | 5.3 | 1.9 | 47 | 21.8 | 27 |
| jco | 1.0 | 0.6 | 31 | 9.1 | 20 | 5.5 | 3.1 | 35 | 12.9 | 31 |
| jox | 1.0 | 0.7 | 45 | 17.2 | 33 | 5.1 | 3.3 | 50 | 26.4 | 44 |
| jph | 1.0 | 0.6 | 36 | 15.9 | 21 | 5.2 | 2.2 | 48 | 21.2 | 38 |
| jth | 1.0 | 0.5 | 28 | 8.0 | 15 | 6.0 | 2.5 | 36 | 9.5 | 29 |
| oeu | 1.0 | 0.4 | 45 | 12.2 | 17 | 5.0 | 2.0 | 49 | 18.9 | 32 |
| pha | 2.4 | 2.3 | 30 | 13.6 | 30 | 2.7 | 2.5 | 46 | 20.7 | 46 |
| pni | 1.6 | 1.5 | 24 | 8.1 | 24 | 2.2 | 2.0 | 39 | 13.2 | 38 |
| ppa | 1.8 | 1.3 | 33 | 13.6 | 25 | 2.3 | 2.2 | 47 | 13.9 | 47 |
| ppe | 1.3 | 1.0 | 24 | 7.9 | 17 | 1.5 | 1.2 | 48 | 16.8 | 38 |
| psy | 1.9 | 1.9 | 18 | 6.0 | 18 | 2.5 | 2.4 | 26 | 8.1 | 26 |
| pun | 1.6 | 0.8 | 5 | 1.2 | 2 | 2.4 | 2.0 | 5 | 1.3 | 5 |
| pav | 1.0 | 0.0 | 33 | 13.3 | 1 | 5.4 | 0.0 | 43 | 15.9 | 1 |
| qca | 1.0 | 0.7 | 9 | 2.4 | 6 | 1.3 | 1.1 | 18 | 3.2 | 18 |
| qco | 1.0 | 0.1 | 33 | 13.3 | 2 | 5.3 | 0.5 | 47 | 23.9 | 13 |
| qfa | 2.3 | 1.9 | 38 | 14.7 | 36 | 2.9 | 2.6 | 45 | 19.5 | 43 |
| qil | 2.8 | 2.2 | 44 | 28.1 | 40 | 3.5 | 3.4 | 50 | 27.8 | 50 |
| qpe | 2.2 | 1.6 | 11 | 4.6 | 9 | 2.9 | 2.5 | 19 | 7.8 | 19 |
| qpu | 2.1 | 0.9 | 7 | 5.0 | 4 | 2.2 | 0.6 | 17 | 3.8 | 6 |
| qpy | 2.2 | 1.5 | 26 | 10.4 | 21 | 2.5 | 2.3 | 44 | 15.5 | 43 |
| qro | 2.5 | 1.8 | 12 | 5.0 | 9 | 2.8 | 2.1 | 15 | 4.9 | 13 |
| qsu | 1.7 | 1.0 | 34 | 11.2 | 18 | 2.6 | 2.2 | 45 | 16.2 | 40 |
| sar | 1.0 | 0.5 | 31 | 6.8 | 17 | 5.6 | 2.6 | 33 | 9.9 | 29 |
| sau | 1.0 | 0.6 | 22 | 8.3 | 14 | 5.7 | 2.7 | 29 | 10.5 | 22 |
| tga | 1.0 | 0.2 | 28 | 11.2 | 5 | 5.1 | 1.0 | 49 | 18.8 | 20 |
| tba | 1.0 | 0.7 | 26 | 12.5 | 19 | 5.6 | 2.3 | 40 | 13.0 | 34 |
| tco | 1.0 | 0.2 | 14 | 5.4 | 3 | 5.7 | 0.4 | 40 | 10.7 | 11 |
| tpl | 1.0 | 0.4 | 18 | 5.8 | 7 | 6.0 | 1.8 | 23 | 8.7 | 19 |
| ugl | 1.0 | 0.4 | 21 | 5.6 | 9 | 5.4 | 2.0 | 27 | 10.6 | 21 |
| umi | 1.0 | 0.5 | 46 | 21.8 | 22 | 5.2 | 2.9 | 49 | 23.2 | 40 |

**Tgp_loc**: Total richness of strictly local genetic pool; **Agp_loc**: Available richness of strictly local genetic pool, **Tdr_loc**: Total number of deployment zones for the strictly local genetic pool; **Edr_loc**: Effective number of deployment zones for the strictly local genetic pool; **Adr-loc**: Number of deployment zones for the available strictly local genetic pool; **Tgp_cloc**: Total richness of Wide-sense local genetic pool; **Agp_cloc**: Available richness of Wide-sense local genetic pool; **Tdr_cloc**: Total number of deployment zones for the Wide-sense local genetic pool; **Edr_cloc**: Effective number of deployment zones for the Wide-sense local genetic pool;**Adr-cloc**: Number of deployment regions for the climate- local genetic pool;
