# Supplementary material for: Local seed sourcing for sustainable forestry
Source: PLoS One. 2022 Dec 14;17(12):e0278866. doi: 10.1371/journal.pone.0278866 (PMC9750025; doi:10.1371/journal.pone.0278866)
Supplement: S3 Table — (DOCX) [file pone.0278866.s005.docx]

**S3 Table**. Data on the use of forest reproductive material of 40 forest tree species in Spain.

| **Species** | **regproc** | ***spa*** | **aff_su** | **aff_pu** | **aff_pr** | **frm_si** | **frm_qt** | **recgen** |
| --- | --- | --- | --- | --- | --- | --- | --- | --- |
| **Code** | # | # | ha/yr | % | % | x1000 plants | x1000 plants | **%** |
| aal | 6 | 1.8 | 0.2 | 100.0 | 100.0 | 8.3 | 0.0 | 50.0 |
| api | 3 | 1.7 | 0.0 | 0.0 | 0.0 | 19.8 | 0.0 | 33.3 |
| apl | 7 | 0.3 | 0.0 | 0.0 | 0.0 | 8.9 | 0.0 | 0.0 |
| aps | 19 | 2.2 | 5.6 | 37.2 | 31.7 | 82.5 | 0.0 | 25.0 |
| aun | 47 | 3.4 | 4.5 | 98.1 | 100.0 | 150.2 | 0.0 | 0.0 |
| bpu | 23 | 0.3 | 47.1 | 24.2 | 35.8 | 262.6 | 3.2 | 15.4 |
| csa | 37 | 10.4 | 288.6 | 20.5 | 25.6 | 199.8 | 46.8 | 0.0 |
| fsy | 18 | 16.1 | 88.4 | 55.0 | 80.4 | 273.6 | 0.0 | 11.1 |
| fex | 17 | 4.6 | 4.5 | 57.3 | 45.9 | 341.4 | 0.0 | 0.0 |
| iaq | 30 | 5.1 | 4.7 | 89.2 | 89.5 | 102.1 | 0.1 | 0.0 |
| jre | 39 | 1.5 | 18.8 | 15.9 | 34.9 | 119.1 | 2.5 | 0.0 |
| jco | 31 | 6.5 | 0.6 | 87.3 | 100.0 | 31.7 | 0.0 | 0.0 |
| jox | 45 | 5.8 | 12.5 | 95.0 | 100.0 | 78.7 | 0.0 | 0.0 |
| jph | 36 | 3.0 | 10.9 | 100.0 | 100.0 | 156.1 | 0.0 | 0.0 |
| jth | 28 | 6.9 | 11.3 | 98.8 | 92.0 | 268.1 | 0.0 | 0.0 |
| oeu | 45 | 1.2 | 54.7 | 29.3 | 98.1 | 106.2 | 0.0 | 0.0 |
| pha | 20 | 15.8 | 551.8 | 80.7 | 92.8 | 1467.2 | 27.6 | 0.0 |
| pni | 15 | 15.2 | 480.9 | 60.5 | 65.5 | 2977.1 | 43.7 | 0.0 |
| ppa | 29 | 11.6 | 2784.6 | 41.4 | 26.6 | 2523.0 | 281.4 | 17.9 |
| ppe | 12 | 6.3 | 457.0 | 58.5 | 58.3 | 4035.8 | 5.0 | 16.7 |
| psy | 19 | 13.1 | 1217.8 | 59.0 | 61.4 | 5756.9 | 249.9 | 23.8 |
| pun | 6 | 6.2 | 42.2 | 100.0 | 100.0 | 1797.4 | 8.0 | 20.0 |
| pav | 33 | 5.9 | 43.4 | 25.8 | 27.6 | 319.3 | 0.0 | 0.0 |
| qca | 5 | 3.0 | 0.0 | 0.0 | 0.0 | 1.7 | 0.0 | 20.0 |
| qco | 33 | 5.3 | 31.4 | 54.8 | 80.6 | 2511.5 | 0.0 | 0.0 |
| qfa | 27 | 9.6 | 46.6 | 86.4 | 87.3 | 13600.6 | 0.0 | 38.5 |
| qil | 28 | 24 | 725.1 | 50.8 | 56.1 | 17216.2 | 0.0 | 35.7 |
| qpe | 13 | 7.6 | 46.7 | 91.2 | 78.7 | 213.7 | 0.0 | 28.6 |
| qpu | 6 | 3.5 | 0.1 | 9.5 | 9.5 | 553.3 | 0.0 | 62.5 |
| qpy | 28 | 9.4 | 84.8 | 92.0 | 92.2 | 4931.0 | 0.0 | 28.6 |
| qro | 11 | 10.8 | 120.5 | 48.6 | 34.0 | 4019.1 | 0.0 | 16.7 |
| qsu | 25 | 9.9 | 814.9 | 54.1 | 81.1 | 2046.8 | 0.0 | 23.1 |
| sar | 31 | 4.8 | 5.6 | 100.0 | 100.0 | 263.2 | 0.0 | 0.0 |
| sau | 22 | 7.0 | 5.3 | 97.9 | 97.2 | 1104.1 | 0.0 | 0.0 |
| tga | 28 | 0.5 | 12.9 | 47.4 | 48.4 | 1749.4 | 0.0 | 0.0 |
| tba | 26 | 3.5 | 5.1 | 86.2 | 88.2 | 68.1 | 0.0 | 0.0 |
| tco | 14 | 0.4 | 0.2 | 100.0 | 100.0 | 247.5 | 0.0 | 12.5 |
| tpl | 18 | 2.0 | 0.3 | 100.0 | 88.3 | 135.2 | 0.0 | 5.3 |
| ugl | 21 | 1.1 | 0.0 | 0.0 | 0.0 | 55.4 | 0.0 | 4.5 |
| umi | 46 | 1.6 | 0.1 | 100.0 | 100.0 | 62.0 | 0.2 | 0.0 |

^1^ ***regpro*** number of regions of provenance of the species; ***spa:*** seed production area (unmber); ***aff_su:*** afforested area/year in ha; ***aff_pu:*** ratio of public afforestation to total afforested area; ***aff_yi:*** ratio of productive afforested area to the total afforested area; ***aff_pr:*** ratio afforested area for protection to the total afforested area; ***frm_si***: source-identified and selected FRM by year in nb of plants; ***frm_qt***: qualified and tested FRM by year in nb of plants
