# Supplementary material for: Local seed sourcing for sustainable forestry
Source: PLoS One. 2022 Dec 14;17(12):e0278866. doi: 10.1371/journal.pone.0278866 (PMC9750025; doi:10.1371/journal.pone.0278866)
Supplement: S4 Table — (DOCX) [file pone.0278866.s006.docx]

**S4 Table.** T-test for different variables related to the genetic pools and use of forest reproductive material of the species, comparing species with regions of provenance delineated using the divisive *vs*. agglomerative methods.

| **Variable** | **Divisive** | **Agglomerative** | **t** |
| --- | --- | --- | --- |
| **Tgp_loc** | 1 (0) | 2.18 (0.55) |  |
| **Agp_loc** | 0.46 (0.21) | 1.72 (0.65) | 7.70*** |
| **Edr_loc** | 9.93 (5.16) | 8.67 (6.81) | 0.66ns |
| **Tdr_loc** | 28.54 (11.44) | 20.44 (13.17) | 2.07* |
| **Adr_loc** | 11.93 (8.58) | 17.12 (13.70) | 1.20ns |
| **Tgp_cloc** | 5.03 (1.14) | 2.45 (0.54) | 9.50*** |
| **Agp_cloc** | 2.06 (1.03) | 2.14 (0.66) | 0.28ns |
| **Edr_cloc** | 14.02 (6.75) | 11.3 (7.87) | 1.17ns |
| **Tdr_cloc** | 36.08 (11.64) | 30 (16.88) | 1.35ns |
| **Adr-cloc** | 24.96 (12.31) | 28 (16.72) | 0.66ns |
| **regproc** | 28.38 (11.76) | 16.63 (8.95) | 3.44** |
| **spa** | 3.6 (2.66) | 10.16 (5.88) | 4.77*** |
| **aff_su** | 23.67 (58.62) | 466.35 (717.75) | 2.53* |
| **aff_pu** | 61.04 (38.61) | 61.73 (29.49) | 0.01ns |
| **aff_pr** | 65.99 (37.57) | 63.99 (31.63) | 0.17ns |
| **dr-loca** | 13.71 (8.58) | 17.13 (11.93) | 1.08ns |
| **frm_si** | 351.03 (600.22) | 3839.99 (4902.87) | 2.92** |
| **frm_qt** | 2.2 (9.53) | 38.48 (89.71) | 1.661NS |
| **recgen** | 3.45 (7.17) | 25.41 (16.5) | 5.14*** |

**Tgp_loc**: Total richness of strictly local genetic pool; **Agp_loc**: Available richness of strictly local genetic pool, **Edr_loc**: Effective number of deployment zone for the strictly local genetic pool; **Tdr_loc**: Total number of deployment zones for the strictly local genetic pool; **Adr-loc**: Number of deployment zones for the available strictly local genetic pool; **Tgp_cloc**: Total richness of Wide-sense local genetic pool; **Agp_cloc**: Available richness of Wide-sense local genetic pool; **Edr_cloc**: Effective number of deployment zones for the Wide-sense local genetic pool; **Tdr_cloc**: Total number of deployment zones for the Wide-sense local genetic pool; **Adr-cloc**: Number of deployment zones for the climate- local genetic pool; **regpro**: number of region of provenances of the species ; **spa**: mean number of seed production areas by region of provenance; **aff_su**: afforested area/year in ha; **aff_pu**: ratio of public afforestation to total afforested area; **aff_pr**: ratio of protection afforested area to the total; **frm_si**: source-identified and selected FRM by year in nb of plants; **frm_qt**: qualified and tested FRM by year in nb of plants, **recgen**: percent of deployment zones with endangered local populations per species.
