# Supplementary material for: Local seed sourcing for sustainable forestry
Source: PLoS One. 2022 Dec 14;17(12):e0278866. doi: 10.1371/journal.pone.0278866 (PMC9750025; doi:10.1371/journal.pone.0278866)
Supplement: Attachment — Submitted filename: Edited_Manuscript.docx [file pone.0278866.s007.docx]

Local seed sourcing for sustainable forestry

Ricardo Alía^1*^, Eduardo Notivol^2^, José Climent^1^, Felipe Pérez^3^, Diana Barba^1^, Juan Majada^4^, José Manuel García del Barrio^1^

^1^ Department of Ecology and Forest Genetics, Institute of Forest Sciences. INIA-CSIC. Madrid. Spain

^2^ Department of Environment, Agricultural and Forest Systems. CITA. Zaragoza. Spain

^3^ Directorate General of Biodiversity, Forest and Desertification. MITECO. Madrid. Spain

^4^ CETEMAS. Carbayin, Spain

* Corresponding author

e-mail: [alia@inia.csic.es](mailto:alia@inia.csic.es) (RA)

**Abstract**

Seed sourcing strategies are the basis for identifying genetic material meeting the requirements of future climatic conditions and social demands. Local seed sourcing has been extensively promoted, based in the expected adaptation of the populations to the local conditions, but there are some limitations for the application. Therefore, we need to analyze these limitations and how to overcome them. We analyzed Strict-sense local and Wide-sense local seed sourcing strategies. We obtained four pools for each strategy (the total and available pools for the species and genetic levels) for 40 species and 50 deployment zones in Spain. We also obtained the total number of seed sources and stands for those species in the EU countries. We analyze the richness of the pools, the relationship with variables related to the use of the species in afforestation, and the availability of basic material approved for the production of forest reproductive material which is to be marketed. This study confirms the existence of extensive species and genetic local pools. In addition to, species differs in the importance of these pools, with limitations derived from the existence of basic materials, and the use of forest reproductive material. Conservative strategies derived from local seed sourcing strategies are the basis for the use of forest reproductive material due to a large number of species and region of provenances under regulation in the area considered in the study. Despite the extensive work done for approving basic materials, some limitations are found in the species under regulation to provide local seed sources for sustainable forestry. We should provide alternative methods to local seed sourcing in order to meet the social demands under the actual regulations on the marketing of reproductive materials.

**Keywords**:

Reforestation, restoration, seed transfer, seed deployment, local provenances

**Introduction**

Sustainable forest management aims at maintaining the biodiversity, productivity, regeneration capacity, vitality and potential of forests (Resolution H1, Forest Europe 1993), taking into account the economic values of ecosystem services they provide [1], and the urgent need to increase the resilience of forests [2].

In this context, both artificial and natural regeneration play essential roles to ensure resilience [3] and long-term persistence [4] of the populations, conserving genetic resources [5], enhancing genetic diversity that can ensure future adaptation [6] and restoring functional ecosystems [7,8].

While natural regeneration is always based on local genetic resources, artificial regeneration often makes use of the deliberate transfer of forest reproductive materials (i.e. seeds, fruits, plants or parts of plants). In this last case, seed sourcing involves the matching of the area from which the reproductive material is obtained (procurement zone) and the area where the material will be used (deployment zone) [9]. The election of the genetic background of planting stock is a major concern in forestry since it influences future performance, as extensive provenance research has repeatedly shown[10].

The election of the local provenance is based on the expectation that populations are locally adapted [11–13], as the origin also influences different traits related to the stability, adaptation, resistance, productivity and diversity of the planting stock [13,14]. This is the most widespread approach [15] and the preferred one in biodiversity conservation [16]. Local seed sourcing is recommended as a general principle in Forest Europe and is followed by many different countries [3]. However, local seed sourcing can be interpreted as strict-sense i.e using material collected from the same region of provenance (i.e. procurement zone according to EU regulations) than the deployment zone or at gene flow distance, or wide-sense, i.e. considering the climatic similarity between the region of provenance and deployment zones) [15,17].

Other strategies have been suggested under climate change scenarios: climate-predictive, composite, admixture, climate-adjusted, and assisted migration [15,18]. Those methods require information on the performance of the planting stock under different environments [19–26].

Based on a seed-sourcing approach, it is possible to define a species pool, i.e. the set of species that can potentially inhabit a site because of suitable local ecological conditions [27], and the genetic pool within those species [28], i.e. the set of genetic materials suitable for the site. The available genetic pool includes the reproductive material meeting the existing regulations [29,30], and therefore the availability of the desired material represents a major limitation in some cases, like ecosystem restoration [31].

We study the application of local seed sourcing for source-identified and selected reproductive materials (EU and OCDE schemes) in the context of sustainable forestry. These categories present some advantages for multipurpose forestry and ecological restoration activities, linked to the precise identification of the origin of the material, high population size and low selection of the material. They also represent 79.2% of the total approved basic material in the EU. We used Peninsular Spain and the Balearic Islands as a model, and we expand our study to the analysis of the available basic material in the EU. Our study area presents a higher tree diversity compared to other areas in Europe [32], as a well-known biodiversity hot-spot with a strong interest in species conservation [33]. In this area, there is a long tradition of multi-purpose forest plantations with different goals [34]: restoration, protection and, to a lesser extent, production [35]. There is a preference for source-identified and selected reproductive materials, from a highly diverse species pool [36]. Source-identified materials represent 82 and 85% of the total reproductive material produced annually in Spain, while selected materials represent 14 to 17%, and qualified and tested represent less than 1% for all the species considered.

In this work, we evaluate two seed-sourcing strategies: Strict-sense local and Wide-sense local (i.e. based on climate similarity). The analysis is based on the regions of provenance and deployment zones, intending to work at the landscape level, and considering the reported scale of gene flow in forest tree species [37]. We analysed 40 native forest tree species with the actual trading of reproductive materials in Spain, and we identified the regions of provenance climatically suitable for each deployment zone for each species. We also defined the potential availability of basic materials based on the existence of approved basic materials (source-identified and selected categories). We computed the richness of pools –at two genetic levels and considering the potential availability– for each of the two seed-sourcing strategies, per species and deployment zone. Our objectives are to determine: i) the richness of those pools per species and deployment zone, ii) climatic trends on the richness of these pools, iii) the relationship among the richness of the pools, the use of reproductive material of the species and the method for defining regions of provenance, and iv) the restrictions due to availability of basic materials. We further discuss the application of local seed-sourcing for afforestation, restoration and reforestation, in highly diverse environments such as the Mediterranean forests, and the application in other areas in Europe in a context of sustainable forestry.

**Material and methods**

We compared the richness and deployment of seed sourcing pools from two strategies: Strict-sense local and Wide-sense local (the last based on climate similarity) for source-identified and selected reproductive materials. All materials considered in the study corresponded to autochthonous populations of 40 species in Spain. We also compared the pool of basic material in the EU for these same species.

**Species and pools**

The 40 studied species (Table 1) are regulated for marketing and trading of forest reproductive material. The species were classified depending on the main use for restoration, protection and to a lesser extent in productive plantations according to published national guidelines [35].

**Table 1. Species considered in the study.**

| **Code^1^** | **Species** | **Use^2^** | **Regulation^3^** | ***regpro***^4^ | **Code** | **Species** | **Use** | **Regulation** | ***regpro*** |
| --- | --- | --- | --- | --- | --- | --- | --- | --- | --- |
| aal | *Abies alba* Mill. | RE | EU | 6^a^ | psy | *Pinus sylvestris* L. | PR,PT | EU | 19^a^ |
| api | *Abies pinsapo* Boiss. | RE | EU | 3^a^ | pun | *Pinus uncinata* Ram. ex DC. | PT | SP | 6^a^ |
| apl | *Acer platanoides* L. | RE | EU | 7^d^ | pav | *Prunus avium* L. | PR,RE | EU | 33^d^ |
| aps | *Acer pseudoplatanus* L. | RE | EU | 19^d^ | qca | *Quercus canariensis* Willd. | RE | SP | 5^a^ |
| aun | *Arbutus unedo* L. | RE | SP | 47^d^ | qco | *Quercus coccifera* L. | RE | SP | 33^d^ |
| bpu | *Betula pubescens* Ehrh. | RE | EU | 23^d^ | qfa | *Quercus faginea* Lam. | RE | SP | 27^a^ |
| csa | *Castanea sativa* Mill. | PR | EU | 37^d^ | qil | *Quercus ilex* L. | PR,RE | EU | 28^a^ |
| fsy | *Fagus sylvatica* L. | PT | EU | 18^a^ | qpe | *Quercus petraea* (Matt.) Liebl. | RE | EU | 13^a^ |
| fex | *Fraxinus excelsior* L. | RE | EU | 17^d^ | qpu | *Quercus pubescens* Willd. | RE | EU | 6^a^ |
| iaq | *Ilex aquifolium* L. | RE | SP | 30^d^ | qpy | *Quercus pyrenaica* Willd. | RE | SP | 28^a^ |
| jre | *Juglans regia* L. | PR | SP | 39^d^ | qro | *Quercus robur* L. | RE | EU | 11^a^ |
| jco | *Juniperus communis* L. | RE | SP | 31^d^ | qsu | *Quercus suber* L. | PR,RE | EU | 25^a^ |
| jox | *Juniperus oxycedrus* L. | RE | SP | 45^d^ | sar | *Sorbus aria* (L.) Crantz | RE | SP | 31^d^ |
| jph | *Juniperus phoenicea* L. | RE | SP | 36^d^ | sau | *Sorbus aucuparia* L. | RE | SP | 22^d^ |
| jth | *Juniperus thurifera* L. | PT | SP | 28^d^ | tga | *Tamarix gallica* L. | RE | SP | 28^d^ |
| oeu | *Olea europea* Brot. | RE | SP | 45^d^ | tba | *Taxus baccata* L. | RE | SP | 26^d^ |
| pha | *Pinus halepensis* Mill. | PT | EU | 20^a^ | tco | *Tilia cordata* Mill. | RE | EU | 14^d^ |
| pni | *Pinus nigra* Arn. | PR,PT | EU | 15^a^ | tpl | *Tilia platyphyllos* Scop. | RE | EU | 18^d^ |
| ppa | *Pinus pinaster* Aiton. | PR,PT | EU | 29^a^ | ugl | *Ulmus glabra* Huds. | RE | SP | 21^d^ |
| ppe | *Pinus pinea* L. | PR,PT | EU | 12^a^ | umi | *Ulmus minor* Mill. s.l. | RE | SP | 46^d^ |

^1^**Code**: According to the Commission Regulation (EC) No 1597/2002 of 6 September 2002. ^2^**Use:** from Peman et al. (2013). RE: restauration, PR: production, PT: protection. ^3^**Regulation** marketing of reproductive material, EU: Council EU Directive 199/105, SP: Spanish RD289/2003., ^3^**regpro**: number of regions of provenance of the species (^a^Agglomerative method, ^d^Divisive method).

Distribution data were available for each species from the Spanish National Inventory and Spanish Forest Map (1/25000) and transformed into species presence/absence data in a grid of 1x1 km^2^. We considered natural populations in the Spanish Iberian Peninsula and Balearic Islands, excluding plantations, according to the regions of provenance of each species [38].

*Deployment zones* were established by a division of Spain in continuous regions with similar environmental characteristics. The method resulted in fifty units for the Iberian Peninsula and Balearic Islands [39,40].

*Regions of provenance,* equivalent to the procurement zones for the seed sourcing schemes. According to the EU regulation [29] they constitute the basic units for source- identified and selected materials. The regions were defined for all the species considered in this study (Table 1) using two different methodologies [38]: An agglomerative method (see eg [41]), was used to group populations of each species with similar ecological, phenotypic or genetic characteristics. These species-specific regions of provenance were defined for the main forest species 17 species of *Abies* (2)*,* *Fagus* (1), *Pinus* (6) and *Quercus* (8) genus). A divisive method was applied for the remaining 23 species. Those regions of provenance were not species-specific and coincident with deployment zones.

**Seed sourcing strategies**

*Strict-sense* *local seed-sourcing strategy* (SSL) was defined for each deployment zone as the use of autochthonous populations pertaining to the same region of provenance for each species. *Wide-sense local* *seed-sourcing strategy* (WSL) was defined as the use of the pool of regions of provenance with a suitable climatic niche in each zone.

Niche modelling was used to obtain the climate-predicted *distribution* of the species based on the actual presence and the climate (assuming no restrictions to dispersal and no human influence) (see [36] and S1 Annex). A similarity index [42] was obtained based on the Mahalanobis climatic distance (using the same set of climatic variables that in the niche modelling estimation, see annex 1), among the points with presence of the region of provenances and the points of each deployment zone. Based on this similarity the regions of provenance were classified as suitable or not for each deployment zone.

A dataset was created including the regions of provenance identified for each of the 40 species and 50 deployment zones using the SSL and WSL strategies.

**Seed sourcing pools**

We defined eight seed-sourcing pools from this database by combining the two seed-sourcing strategies SSL and WSL at two levels (species and genetic) and considering the availability (yes/no) of basic material in the national register.

The *species pool* was defined as the set of species for each deployment zone following each of the two seed-sourcing strategies. Similarly, the *genetic pool* was defined as the set of regions of provenance defined by each of the strategies.

The *available pool* was defined as those regions of provenance with at least one basic material in the national register (source-identified or selected categories). The National Register held by the National Authority includes the existing approved basic materials (<https://www.miteco.gob.es/es/biodiversidad/temas/recursos-geneticos/geneticos-forestales/rgf_catalogo_materiales_base.aspx>; data accessed 31/12/2021).

**Data on FRM and use of the species**

The actual use of the species in afforestation and reforestation programs was defined by the following variables: 1. Number of region of provenances of the species (*regpro*); 2. Mean number of basic material (entries or accessions in the national catalogue) by region of provenance (basic_mat); 3. Afforested area/year in ha (*aff_su*); 4. Ratio of public afforestation to total afforested area (*aff_pu*); 5. Ratio afforested area for protection to total afforested area (*aff_pr*); 6. Source-identified and selected FRM by year in number of plants (*frm_si*) and 7. Qualified and tested FRM by year in number of plants (*frm_qt*). Data on afforestation and production of FRM was obtained as the mean for the period 2005-2016 (last year with available data) based on the Spanish national forestry statistics.

We also calculated: 8. The percent of deployment zones with endangered local populations (*recgen*) for each species, following the criteria in Spanish Strategy for the Conservation and Sustainable Use of Forest Genetic Resources [43][38]. This value was used as a proxy to the genetic risk for transferring materials to a given zone.

**Pool of basic material by Country in the EU**

We computed the available pool of basic material compiled for each EU country, and for the species in our study included in the FOREMATIS database (<https://ec.europa.eu/forematis/>) by category (source-identified, selected, qualified and tested).

**Statistical Analysis**

**Richness of seed sourcing pools per deployment zone**

We obtained the richness of the eight pools already defined for each deployment zone and we analyzed the climatic factors related to the richness increment between Strict-sense and Wide-sense local for the different categories (species, genetic and available genetic). A stepwise selection model was applied to start with a saturated model with the eight standardized variables and remove the least significant variables until no further decrease in the Bayesian Information Criterion (BIC). Non-significant variables were removed from the final model. Eight climatic variables were considered: mean value of Altitude (ALT, m), Rainfall (P, mm), summer precipitation (SP, mm), Dry period (DP, months), Frost period (FP, months), Annual mean temperature (TM, Celsius degrees), Mean of the Maximum temperatures of the hottest month (MXHM, Celsius degrees) and Mean of the minimum temperatures of the coldest month (*MNCM*, Celsius degrees).

**Relationship among seed sourcing pools and use of the species**

We explored the relationship among the set of variables describing the seed sourcing pools by species (richness of the genetic and available genetic pools, number and harmonic mean of deployment zones -equivalent to the effective number of deployment zones- according to the frequency of the species’ present in the region), and the variable set describing the actual deployment of reproductive materials (see above). A canonical correspondence analysis (CCA) [44], was performed and the biplot of variables and species was used to explore the relationship among these two sets of variables.

A T-test for two means with unknown population standard deviations was applied to explore the relationship among the variables related to the seed sourcing pools and the use of the species for the two main classes of species: those with regions of provenance defined by an agglomerative method and the ones defined by using a divisive method.

**EU Trends of basic materials**

We computed the number of basic materials (source-identified and selected categories) or the set of 40 species, and the harmonic mean (equivalent to effective number per species) for each country and for the sum of source-identified and selected materials, and qualified and tested materials.

We compared by ANOVA the values of the number of species under regulation in each country from the 40 considered in the study, and the mean values for the source-identified, selected and qualified-tested material for different EU regions (Nordic, Western Central, Eastern Central, Western South, Eastern South).

All statistical analyses were made within the R environment (R Core Team, 2015), with the packages corrplot [45], Hmisc [46], CCA [44], and MASS [47].

**Results**

**Seed sourcing pools by deployment zone**

The species differed in their range of distribution, and therefore in the local materials available for each deployment zone. The richness of the species pool (for both SSL and WSL strategies) varied greatly among deployment zones. The mean value of the SSL species pool was 20.2 ± 7.6 (range from 4 to 36 species) with a slight increment for the WSL species pool to 26.9 ± 8.1 (range from 8 to 39) (Fig 1, S1 Table).

**Fig 1.** **Richness of pools for Strict-sense and Wide-sense local seed-sourcing strategies for the species (A, B), genetic (C, D) and available genetic pools (E, F).**

When considering the genetic pools, richness increased greatly for both seed-sourcing strategies: to 27.7 ± 12.6 (range from 4 to 63) for SSL and to 114.1±57.8 (range from 10 to 245) for WSL.

However, the available genetic pool, (basic materials in the national register), for all species, decreased to 17.7 ± 10.1 (60.2% of the total) for SSL and 57.6 ± 33.9 (48.4% of the total) for WSL strategy.

The increment of richness among SSL and WSL pools showed a climatic trend associated mainly to temperature for the three levels considered: species, genetic and available genetic (Table 2), with a reduction in the more thermic areas, in southern Spain.

**Table 2. Regression analysis for the increment of richness for different seed sourcing pools (Strict-sense local and Wide-sense local) per deployment zone and climatic variables. Variables retained after a stepwise selection model starting with the eight standardized variables:**

|  |  |  |  |  | **Analysis of variance** | | |
| --- | --- | --- | --- | --- | --- | --- | --- |
|  | **Estimate** | **Std. Error** | **t-value** | **Pr(>\|t\|)** | **M. Squares** | **F-value** | **Pr(>F)** |
| ***Species pool*** | | | | | | | |
| ALT | -23.642 | 0.9222 | -2.564 | 0.014025 | 31.276 | 7.1488 | 0.010640 |
| P | 19.680 | 0.5541 | 3.551 | 0.000961 | 42.224 | 9.6509 | 0.003387 |
| MXHM | -39.996 | 0.7162 | -5.585 | 1.57e-06 | 42.865 | 9.7974 | 0.003175 |
| MNCM | -59.040 | 11.896 | -4.963 | 1.20e-05 | 107.765 | 24.6316 | 1.2e-05 |
| ***Genetic pool*** | | | | | | | |
| P | -14.999 | 7.379 | -2.033 | 0.04801 | 1080 | 0.9254 | 0.341203 |
| SP | -17.473 | 9.397 | -1.859 | 0.06952 | 12337 | 10.5714 | 0.002179 |
| TM | -76.989 | 11.677 | -6.593 | 4.09e-08 | 47407 | 40.6211 | 8.696e-08 |
| FRO | -29.741 | 9.189 | -3.237 | 0.00227 | 12225 | 10.4754 | 0.002273 |
| ***Available Genetic pool*** | | | | | | | |
| P | -9.846 | 3.645 | -2.702 | 0.00976 | 1671.1 | 6.3874 | 0.015161 |
| TM | -67.208 | 21.459 | -3.132 | 0.00309 | 17157.3 | 65.5788 | 2.918e-10 |
| FRO | -11.473 | 4.402 | -2.606 | 0.01245 | 2188.7 | 8.3658 | 0.005923 |

ALT: Altitude, P: rainfall, TM: Mean annual Temperature, SP: Summer Precipitation, FRO: Frost period, MXHM: Maximum temperature of the hottest month; MNCM: Minimum temperature of the coldest month. Arid Period was not retained for any case

**Seed sourcing genetic pools per species**

The richness of genetic pools per deployment zone differed greatly between SSL and WSL strategies: 1.5±0.7 and 4.0±1.6, respectively. Moreover, the available pools were significantly lower (1.0±0.8 and 2.1±0.9 respectively). For the 40 species, only 41% of the regions of provenance had at least one basic material (source-identified or selected categories) in the national register, with a mean value of 5.8 basic materials per region of provenance (S2 Table).

Reciprocally, the pools can be used in a significantly higher number of deployment zones (133% as an average increment, from 25.3±12.6 in the Strict-sense local to 33.6±14.1 in the Wide-sense local). This value is also reduced when considering availability (15.1±10.0 and 26.2±14.1 respectively).

The canonical correlation analysis showed a trend in the biplot of species on the two first components based on variables related to the use of forest reproductive material, with a transition from those species more extensively used to those used mostly for ecological restoration (Fig 2, S3 Table). The species with an agglomerative method have a lower number of regions of provenance, higher amount of base material, higher afforested surface, and higher production of forest reproductive material, including also a higher number of endangered populations (S4 Table).

**Fig 2. Biplot of the two first components of a CCA analysis on variables related to seed sourcing pools and variables related to the use of FRM for each of the 40 studied species.**

When comparing the pool richness based on the existing basic material for source-identified and selected material in Europe, there were large differences among countries (Fig 3), with higher number of species with basic material and higher number of basic materials in southern counties (Table 3), but still the variability among countries within a region was quite high.

**Table 3. ANOVA for different variables of basic material in EU regions.**

| **Region** | **SP_Forematis** | **SP_study** | **MBA_is** | **SP_qt** | **MBA_qt** |
| --- | --- | --- | --- | --- | --- |
| Northern EU | 20.0 ± 9.63 | 10.2 ± 3.87 | 5.54 ± 3.01 | 6.8 ± 3.06 | 3.09 ± 1.36 |
| Central East EU | 34.0 ± 13.71 | 17.8 ± 3.56 | 9.71 ± 3.93 | 7.8 ± 4.76 | 2.35 ± 1.48 |
| Central West EU | 31.3 ± 14.69 | 15.8 ± 5.39 | 4.88 ± 2.36 | 5.5 ± 5.21 | 1.96 ± 1.17 |
| Southern East EU | 20.3 ± 12.34 | 11.8 ± 9.11 | 4.35 ± 4.75 | 3.5 ± 3.11 | 0.97 ± 0.69 |
| Southern West EU | 52.7 ± 25.81 | 28.7 ± 12.06 | 17.37 ± 13.25 | 3.7 ± 2.52 | 2.22 ± 0.25 |
| Total general | 30.0 ± 16.89 | 15.7 ± 8.1 | 7.32 ± 6.32 | 5.7 ± 4.15 | 2.17 ± 1.27 |
| Anova F test, α | 2.32, 0.081 ns | 3.56, 0.018 * | 3.20, 0.027 * | 1.18, 0.354 ns | 1.56 , 0.216 ns |

**SP_Forematis**: number of species included in the forematis database for each country; **SP_Study**: number of species included in the forematis database for each country included in this study; **MBA_is**: harmonic mean of numer of basic material for the production of forest reproductive material from source-identified and selected categories; **SP_qt**: number of species included in the forematis database for each country with basic material for the production of forest reproductive material of qualified and tested categories (excluding clones and parent of families), **MBA_qt**: harmonic mean of number of basic material for the production of forest reproductive material from qualified and tested categories.

**Fig 3. Basic material in the EU (source www.forematis.eu).**

**Discussion**

We compared the effects of the potential genetic diversity (proxied by the number of suitable and available basic materials) for the reforestation between two alternative seed sourcing strategies for 40 species in Spain.

Our analysis is based on deployment zones and regions of provenance unlike other studies that worked at a finer scale, population level (e.g. [25,48]). Regions of provenance are defined using different criteria among and within countries [49], usually within genetic groups defined by neutral markers [50–52]. For autochthonous or indigenous populations (if the origin is known), a region of provenance can be considered as those populations within gene-flow distance and therefore suitable for local seed-sourcing.

We found that the Strict-sense local species pool was quite high in most of the deployment zones (mean value of 22), with wide genetic pools for those species. We considered Wide-sense local seed sourcing in our study assuming that climate prediction is a good estimate of the performance of the material, *i.e.* the material from ecologically more similar procurement zones would be preferred for a given deployment zone [see 53,54]. This is a more generalized local seed sourcing method than can be implemented for many different species. By using this strategy, the number of basic materials expected to be adapted to the local conditions increased from an average of 1.4 per species and deployment zone to 2.3. This strategy, however, is still rather conservative, discarding recommendations of climatically distant basic materials for a given deployment zone.

We found a highly expectable climatic trend in the richness increment of the pools, associated with higher annual rainfall (or reduction of drought period) and temperatures. This is a general trend for species diversity, where annual precipitation and mean annual temperature play key favorable roles [55]. The species pool seems wide enough for most of the goals of the plantations (restoration, revegetation, afforestation), allowing the establishment of mixed forests with species differing in functional traits [56] in order to obtain multifunctional forests [57], particularly relevant in the Mediterranean region.

Despite the widespread use of Strict-sense local seed sourcing in many countries and regions, there are relevant concerns about this strategy. Local is not always the best option, due to different causes: the absence of native forest species in the deployment zone is an obvious reason. Moreover, low performance or bad adaptation of the local material has also been reported in some cases [58–61], deriving from reduced population size [62], poor seed crops, low genetic diversity (neutral or adaptive), or unfavorable genetic correlations due to trade-offs among traits in highly contrasting ecosystems [63].

Our study also indicates how relevant the availability –linked to the existence of previously approved basic materials– can be as a limiting factor decreasing the potential richness of genetic pools. Despite the important effort in Europe for approving basic materials for many important forest species, still, not all the regions of provenance are listed in the national registers. This drawback makes ecosystem restoration activities to be often constrained by a lack of the desired or suitable basic material [31], legal restrictions for seed collection in endangered populations [64] and. biological constraints such as masting, difficult conservation of seeds and fruits and low population size.

The implementation of alternative seed sourcing strategies (*eg*. assisted migration, admixture, climate-adjusted) to maximize the future adaptation and resilience of our forests [18,61,65,66] is not feasible in the short term for most of the species under consideration in this study as we lack the precise information, or the ecological and evolutionary implications derived from the movement of genetic resources in the landscape [67–69]. An integrative approach aimed at developing suitable restoration materials for a given area [70] based on their out planting performance requires well designed field experiments with representative planting stock quality [71]. On the other hand, the rapid development of genomic research can contribute to optimizing seed sourcing strategies by increasing our knowledge of local adaptation and the main drivers determining the patterns of variation. The existing genetic characterization by molecular markers and/or field trials (comparative common gardens) is clearly insufficient to improve seed sourcing strategies for most of the species. Obtaining this information is costly in money and time (particularly provided robust field experimental settings with multiple sites) since fitness assessment in forest tree species obliges long-term experimentation [72]. We also need to take into consideration that many local populations will not be in suitable areas in the future, as in the case of southern populations of Scots pine [61] or in Maritime pine or Aleppo pine [54]. Therefore,

The pattern of the major importance of source-identified and selected material is a general trend in Europe (84%). In Nordic and Central East European countries the qualified and tested materials increase their importance in the trade of reproductive materials for some of the cited species, and the election of planting stock should be based on *improved /cultivar seed sourcing strategies* [25,73–75] not considered in our study. Source-identified and selected materials could provide material for restoration and sustainable forestry [76], as there are extensive genetic pools in most countries, and these materials have known regions of provenance, large levels of genetic variation [13], and the forest reproductive material have been collected under the supervision of the national authorities. However, the existing differences among countries suggest the necessity of homogenizing information on the characteristics of materials, and deployment zones to facilitate the election by users.

**Conclusions**

Local seed sourcing strategies should be the basis for sustainable forestry until more scientifically contrasted information is available. Strict-sense local and Wide-sense local seed sourcing strategies provide sufficient species and provenance pools for the deployment zones. These pools show a climatic gradient associated with higher annual rainfall and temperatures. The availability of forest reproductive material from specific sources reduces the pools for the different deployment zones, despite the huge efforts in approving basic materials in Spain and the EU in general. Therefore, it would be necessary to increase the planning efforts to meet demand and offer specific seed sources.

**Compliance with ethical standards:**

Not applicable

**Acknowledgements**

This research was supported by the Project AEG 17-048 established in the frame of the measure 15.2“support to the conservation and use of forest genetic resources” and under Regulation (EU) No 1305/2013 of the European Parliament and of the Council of 17 December 2013 on support for rural development by the European Agricultural Fund for Rural Development (EAFRD) with 75% co-financing. This study was funded by the European Union Horizon 2020 research and innovation programme under grant agreement No 773383 (B4EST project), and the Ministry of Science (RTI2018-094691-B-C32).

**Author contributions**

RA; Conceptualization: RA, EN, JC, JM. Formal analysis: RA, EN, JMGB. Data curation: DB. Validation: FP. Writing-original draft preparation: RA. Writing-review and editing: ALL AUTHORS. Funding acquisition, RA, JC, FP.

**Data Availability.** Data is available at https://www.genfored.es/recomendaciones-de-uso/

**References**

1.	Campos P, Caparrós A, Oviedo JL, Ovando P, Álvarez-Farizo B, Díaz-balteiro L, et al. Bridging the gap between national and ecosystem accounting application in andalusianforests, Spain. Ecol Econ. 2019;157: 218–236. doi:10.1016/j.ecolecon.2018.11.017

2.	European_Comssion. New EU Forest Strategy for 2030. COM(2021) 572 Final. 2021; 27.

3.	Konnert M, Fady B, Gömöry D, A , Wolter F, Ducci F, et al. Use and transfer of forest reproductive material in Europe in the context of climate change. Rome, Italy: Bioversity International; 2015. Available:

4.	Ruiz-Jaen MC, Mitchell Aide T, Aide TM. Restoration success: How is it being measured? Restor Ecol. 2005;13: 569–577.

5.	Graudal L, Aravanopoulos FA, Bennadji Z, Changtragoon S, Fady B, Kjær ED, et al. Global to local genetic diversity indicators of evolutionary potential in tree species within and outside forests. For Ecol Manage. 2014;333: 35–51. doi:10.1016/j.foreco.2014.05.002

6.	Fady B, Cottrell J, Ackzell L, Alía R, Muys B, Prada A, et al. Forests and global change: what can genetics contribute to the major forest management and policy challenges of the twenty-first century? Reg Environ Chang. 2016;16: 927–939. doi:10.1007/s10113-015-0843-9

7.	Stanturf JA. What is forest restoration? In: Stanturf, J.A., Madsen P, editor. Restoration of Boreal and Temperate Forests. Boca Raton: CRC Press; 2005. pp. 3–11.

8.	Kettenring KM, Mercer KL, Reinhardt Adams C, Hines J. Application of genetic diversity-ecosystem function research to ecological restoration. J Appl Ecol. 2014;51: 339–348. doi:10.1111/1365-2664.12202

9.	van Buijtenen JP. Fundamental Genetic Principles. In: Fins AT, Brotschol J V, Friedman L, editors. Handbook of Quantitative Forest Genetics. Dordrecht: Kluwer Academic Publishers; 1992. pp. 29–68.

10.	Koskela J, Vinceti B, Dvorak W, Bush D, Dawson IK, Loo J, et al. Utilization and transfer of forest genetic resources: a global review. For Ecol Manage. 2014;333: 22–34. doi:10.1016/j.foreco.2014.07.017

11.	Kawecki TJ, Ebert D. Conceptual issues in local adaptation. Ecol Lett. 2004;7: 1225–1241. doi:10.1111/j.1461-0248.2004.00684.x

12.	Savolainen O, Pyhajarvi T, Knurr T, Pyhäjärvi T, Knürr T. Gene flow and local adaptation in tees. Annu Rev Ecol Evol Syst. 2007;38: 595–619. doi:10.1146/annurev.ecolsys.38.091206.095646

13.	Alberto FJ, Aitken SN, Alía R, González-Martínez SC, Hänninen H, Kremer A, et al. Potential for evolutionary responses to climate change - evidence from tree populations. Glob Chang Biol. 2013;19: 1645–1661. doi:10.1111/gcb.12181

14.	Mátyás C. Climatic adaptation of trees: Rediscovering provenance tests. Euphytica. 1996;92: 45–54.

15.	Breed MF, Stead MG, Ottewell KM, Gardner MG, Lowe AJ. Which provenance and where? Seed sourcing strategies for revegetation in a changing environment. Conserv Genet. 2013;14: 1–10. doi:10.1007/s10592-012-0425-z

16.	IUCN. Afforestation and Reforestation for Climate Change Mitigation: Potentials for Pan-European Action. Quality. Warsaw: IUCN Programme Office for Central Europe; 2004.

17.	Havens K, Vitt P, Still S, Kramer AT, Fant JB, Schatz K. Seed Sourcing for Restoration in an Era of Climate Change. Nat Areas J. 2015;35: 122–133. doi:10.3375/043.035.0116

18.	Prober SM, Byrne M, Mclean EH, Steane DA, Potts BM, Vaillancourt RE. Climate-adjusted provenancing: a strategy for climate-resilient ecological restoration. Front Ecol Environ. 2015;3: 65. doi:10.3389/fevo.2015.00065

19.	Parker WH. Focal point seed zones: site-specific seed zone delineation using geographic information systems. Can J For Res. 1992;22: 267–271.

20.	Wang T, Hamann A, Yanchuk A, O’Neill G a., Aitken SN. Use of response functions in selecting lodgepole pine populations for future climates. Glob Chang Biol. 2006;12: 2404–2416. doi:10.1111/j.1365-2486.2006.01271.x

21.	Lindgren D, Ying CC. A model integrating seed source adaptation and seed use. New For. 2000;20: 87–104.

22.	Wang T, O’Neill G a, Aitken SN. Integrating environmental and genetic effects to predict responses of tree populations to climate. Ecol Appl. 2010;20: 153–63.

23.	Farjat A, Reich BJ, Guinness J, Whetten R, Mckeand S, Isik F. Optimal Seed Deployment under Climate Change using Spatial Models : Application to Loblolly Pine in the Southeastern US. J Am Stat Assoc. 2017;112: 909–920. doi:10.1080/01621459.2017.1292179

24.	Ramalho CE, Byrne M, Yates CJ. A Climate-Oriented Approach to Support Decision-Making for Seed Provenance in Ecological Restoration. Front Ecol Evol. 2017;5: 1–10. doi:10.3389/fevo.2017.00095

25.	Berlin M. Planter’s guide - a joint decision support tool for Scots pine plant material in Sweden and Finland. 2019. Available: https://www.skogforsk.se/english/news/2019/common-scots-pine-deployment-recommendations-for-sweden-and-finland/

26.	Hamann A, Koshy MP, Namkoong G, Ying CC. Genotype-environment interactions in Alnus rubra : developing seed zones and seed-transfer guidelines with spatial statistics and GIS. For Ecol Manage. 2000;136: 107–119.

27.	Zobel M, Maarel E van der, Cecilia D. Species pool: the concept, its determination and significance for community restoration. Appl Veg Sci. 1998;1: 56–66.

28.	Jones TA, Monaco TA. A role for assisted evolution in designing native plant materials for domesticated landscapes. Front Ecol Environ. 2009;7: 541–547. doi:10.1890/080028

29.	Council EU. Council Directive 1999/105/EC of 22 December 1999 on the marketing of forest reproductive material. Off J Eur Communities. 2000;11: 17–40.

30.	Nanson A. The New OECD Scheme for the Certification of Forest Reproductive Materials. Silvae Genet. 2001;50: 181–187.

31.	Jalonen R, Valette M, Boshier D, Duminil J, Thomas E. Forest and landscape restoration severely constrained by a lack of attention to the quantity and quality of tree seed: Insights from a global survey. Conservation Letters Wiley-Blackwell; Jul 1, 2018 pp. 1–9. doi:10.1111/conl.12424

32.	Scarascia-Mugnozza G, Oswald H, Piussi P, Radoglou K. Forests of the Mediterranean region: Gaps in knowledge and research needs. For Ecol Manage. 2000;132: 97–109. doi:10.1016/S0378-1127(00)00383-2

33.	Myers N, Mittlermeier RA, Mittlermeier CG, da Fonseca GA, Kent J. Biodiversity hotspots for conservation priorities. Nature. 2000;403: 853–858. doi:10.1038/468895a

34.	Fernández-Ondoño E, Serrano LR, Jiménez MN, Navarro FB, Díez M, Martín F, et al. Afforestation improves soil fertility in south-eastern Spain. Eur J For Res. 2010;129: 707–717. doi:10.1007/s10342-010-0376-1

35.	Peman J, Navarro-Cerrillo RM, Nicolás JL, Prada MA, Serrada R. Produccion y manejo de semillas y plantas forestales. Vol II. Madrid: OAPN, MAGRAMA; 2013.

36.	García del Barrio JM, Auñón F, Sánchez de Ron D, Alía R. Assessing regional species pools for restoration programs in Spain. New For. 2013;44: 559–576. doi:10.1007/s11056-013-9363-y

37.	Kremer A, Ronce O, Robledo-Arnuncio JJ, Guillaume F, Bohrer G, Nathan R, et al. Long-distance gene flow and adaptation of forest trees to rapid climate change. Ecol Lett. 2012;15: no-no. doi:10.1111/j.1461-0248.2012.01746.x

38.	Alía R, Garcia del Barrio JM, Iglesias S, Mancha JA, de Miguel J, Nicolás JL, et al. Regiones de procedencia de especies forestales de España. Madrid, Spain: O.A. Parques Nacionales; 2009.

39.	García del Barrio JM, de Miguel J, Alía R, Iglesias S. Regiones de identificacion y utilizacion de material forestal de reproduccion. Serie Cartografica. Madrid: MAPA; 2001.

40.	García del Barrio JM, Iglesias S, Alía R. Regiones de Identificación y Utilización de Material Forestal de Reproducción. Regiones de procedencia en España realizadas por el métodod divisivo. Adenda. Madrid: Ministerio de Medio Ambiente; 2005.

41.	Catalan G, Gil P, Galera R, Martín S, Agúndez D, Alía R. Las regiones de procedencia de Pinus sylvestris L. y Pinus nigra Arn subsp. salzmanii (Dunal) Franco en España. Madrid: ICONA; 1991.

42.	Doherty KD, Butterfield BJ, Wood TE. Matching seed to site by climate similarity: Techniques to prioritize plant materials development and use in restoration. Ecol Appl. 2017;27: 1010–1023. doi:10.1002/eap.1505

43.	Jiménez P, Diaz-Fernandez PM, Iglesias S, Prada MA, Garcia del Barrio JM, Alba N, et al. Strategy for the Conservation and Sustainable Use of Spanish Forest Genetic Resources. Inv Agrar Sist y Rec. 2009;18: 13–19.

44.	Gonzalez I, Dejean S, Martin PGP, Baccini A. Journal of statistical software. J Stat Softw. 2008;23: 1–14. doi:10.1002/wics.10

45.	Wei T, Simko V. R package “corrplot”: Visualization of a Correlation Matrix. 2021. Available: https://github.com/taiyun/corrplot

46.	Harrell FE. Hmisc: A package of miscellaneous R functions. 2021. Available: https://cran.r-project.org/package=Hmisc

47.	Ripley B. Support Functions and Datasets for Venables and Ripley’s MASS. 2022. Available: http://www.stats.ox.ac.uk/pub/MASS4/

48.	Lesser MR, Parker WH. Comparison of canonical correlation and regression based focal point seed zones of white spruce. Can J For Res. 2006;36: 1572–1586.

49.	Auñon FJ, Garcia del Barrio JM, Mancha JA, de Vries SMG, Alía R. Regions of provenance of European beech ( Fagus sylvatica L .) in Europe. In: von Wuehlisch G, Alia R, editors. Genetic resources of European beech (Fagus sylvatica L) for sustainable Forestry. Madrid (España): INIA; 2010. pp. 141–148.

50.	Rodríguez-Quilón I, Santos-del-Blanco L, Grivet D, Jaramillo-Correa JP, Majada J, Vendramin GG, et al. Local effects drive heterozygosity–fitness correlations in an outcrossing long-lived tree. Proc R Soc B Biol Sci. 2015;282: 20152230. doi:10.1098/rspb.2015.2230

51.	De Kort H, Mergeay J, Vander Mijnsbrugge K, Decocq G, Maccherini S, Kehlet Bruun HH, et al. An evaluation of seed zone delineation using phenotypic and population genomic data on black alder Alnus glutinosa. J Appl Ecol. 2014;51: 1218–1227. doi:10.1111/1365-2664.12305

52.	Bucci G, Vendramin GG. Delineation of genetic zones in the European Norway spruce natural range: preliminary evidence. Mol Ecol. 2000;9: 923–34.

53.	Serra-Varela MJ, Grivet D, Vincenot L, Broennimann O, Gonzalo-Jiménez J, Zimmermann NE. Does phylogeographical structure relate to climatic niche divergence? A test using maritime pine ( P inus pinaster Ait.). Glob Ecol Biogeogr. 2015;24: 1302–1313. doi:10.1111/geb.12369

54.	Serra-Varela MJ, Alía R, Daniels RR, Zimmermann NE, Gonzalo-Jiménez J, Grivet D. Assessing vulnerability of two Mediterranean conifers to support genetic conservation management in the face of climate change. Divers Distrib. 2017;23: 507–516. doi:10.1111/ddi.12544

55.	Baudena M, Sánchez A, Georg CP, Ruiz-Benito P, Rodríguez MA, Zavala MA, et al. Revealing patterns of local species richness along environmental gradients with a novel network tool. Sci Rep. 2015;5: 1–15. doi:10.1038/srep11561

56.	Baeten L, Verheyen K, Wirth C, Bruelheide H, Bussotti F, Finér L, et al. A novel comparative research platform designed to determine the functional significance of tree species diversity in European forests. Perspect Plant Ecol Evol Syst. 2013;15: 281–291. doi:10.1016/j.ppees.2013.07.002

57.	Manning P, Van Der Plas F, Soliveres S, Allan E, Maestre FT, Mace G, et al. Redefining ecosystem multifunctionality. Nat Ecol Evol. 2018;2: 427–436. doi:10.1038/s41559-017-0461-7

58.	Namkoong G. Nonoptimality of local races. Proceedings of the tenth southern Conference on Forest Tree Improvement. Houston, Texas; 1969.

59.	Leimu R, Fischer M. A Meta-Analysis of Local Adaptation in Plants. PLoS One. 2008;3: 1–8. doi:10.1371/journal.pone.0004010

60.	Jones TA. When local isn’t best. Evol Appl. 2013;6: 1109–1118. doi:10.1111/eva.12090

61.	Notivol E, Santos-Del-blanco L, Chambel R, Climent J, Alía R. Seed sourcing strategies considering climate change forecasts: A practical test in scots pine. Forests. 2020;11: 1–17. doi:10.3390/f11111222

62.	Robledo-Arnuncio JJ, Alía R, Gil LA. Increased selfing and correlated paternity in a small population of a predominantly outcrossing conifer, Pinus sylvestris. Mol Ecol. 2004;13: 2567–77. doi:10.1111/j.1365-294X.2004.02251.x

63.	Ramírez-Valiente JA, Cavender-Bares J. Evolutionary trade-offs between drought resistance mechanisms across a precipitation gradient in a seasonally dry tropical oak (Quercus oleoides). Tree Physiol. 2017;37: 889–901. doi:10.1093/treephys/tpx040

64.	Sánchez-Robles JM, Balao F, García-Castaño JL, Terrab A, Navarro-Sampedro L, Talavera S. Nuclear Microsatellite Primers for the Endangered Relict Fir, Abies pinsapo (Pinaceae) and Cross-Amplification in Related Mediterranean Species. Int J Mol Sci. 2012;13: 14243–50. doi:10.3390/ijms131114243

65.	Crowe KA, Parker WH. Using portfolio theory to guide reforestation and restoration under climate change scenarios. Clim Change. 2008;89: 355–370. doi:10.1007/s10584-007-9373-x

66.	Rehfeldt GE, Ying CC, Spittlehouse DL, Hamilton DA. Genetic responses to climate in Pinus contorta: Niche breadth, climate change, and reforestation. Ecol Monogr. 1999;69: 375–407.

67.	Ahteensuu M, Lehvävirta S. Assisted Migration, Risks and Scientific Uncertainty, and Ethics: A Comment on Albrecht et al.’s Review Paper. J Agric Environ Ethics. 2014 [cited 7 Feb 2014]. doi:10.1007/s10806-014-9493-z

68.	McLachlan JS, Hellmann JJ, Schwartz MW. A framework for debate of assisted migration in an era of climate change. Conserv Biol. 2007;21: 297–302. doi:10.1111/j.1523-1739.2007.00676.x

69.	Hamann A, Roberts DR, Barber QE, Carroll C, Nielsen SE. Velocity of climate change algorithms for guiding conservation and management. Glob Chang Biol. 2015;21: 997–1004. doi:10.1111/gcb.12736

70.	Landis TD, Dumroese RK. Applying the target plant concept to nursery stock quality. Plant Qual - A key to success For Establ Proc CONFORD Conf. 2006; 1–10.

71.	Pinto JR, Dumroese RK, Davis AS, Landis TD. Conducting seedling stocktype trials: A new approach to an old question. J For. 2011;109: 293–299. doi:10.1093/jof/109.5.293

72.	Ramírez-Valiente JA, Santos del Blanco L, Alía R, Robledo-Arnuncio JJ, Climent J. Adaptation of Mediterranean forest species to climate: Lessons from common garden experiments. J Ecol. 2021; 1–21. doi:10.1111/1365-2745.13730

73.	Chakraborty D, Wang T, Andre K, Konnert M, Lexer MJ, Matulla C, et al. Adapting Douglas-fir forestry in Central Europe: evaluation, application, and uncertainty analysis of a genetically based model. Eur J For Res. 2016;135: 919–936. doi:10.1007/s10342-016-0984-5

74.	Freitas ECS de, Paiva HN de, Neves JCL, Marcatti GE, Leite HG. Modeling of eucalyptus productivity with artificial neural networks. Ind Crops Prod. 2020;146. doi:10.1016/j.indcrop.2020.112149

75.	Marcatti GE, Resende RT, Resende MD V., Ribeiro CAAS, dos Santos AR, da Cruz JP, et al. GIS-based approach applied to optimizing recommendations of Eucalyptus genotypes. For Ecol Manage. 2017;392: 144–153. doi:10.1016/J.FORECO.2017.03.006

76.	Alía R, Alba N, Chambel MR, Barba D, Iglesias S. Genetic quality of forest reproductive materials in Land restoration programmes. In: Vallejo VR, Bautista S, Aaronson J, editors. Innovative approaches in forest restoration. Valencia: CEAM; 2009. pp. 89–103.
